# Supplementary material for: Retinal Injury Activates Complement Expression in Müller Cells Leading to Neuroinflammation and Photoreceptor Cell Death
Source: Cells. 2023 Jun 30;12(13):1754. doi: 10.3390/cells12131754 (PMC10340218; doi:10.3390/cells12131754)
Supplement: Supplementary file 1 [file cells-12-01754-s001.zip › cells-2407807-supplementary.pdf]

Supplementary figures

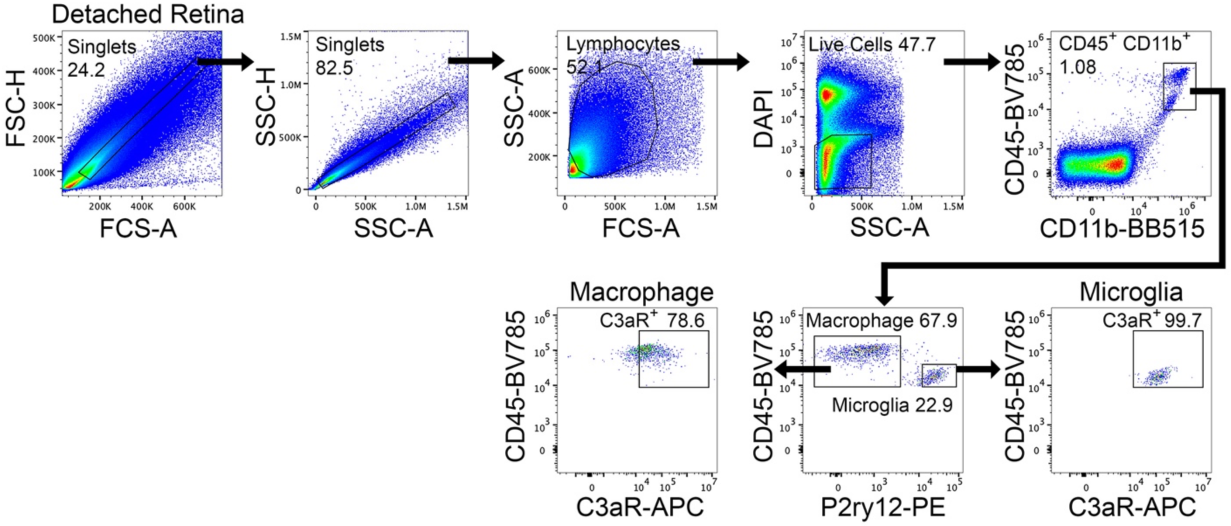

Supplementary figure S1: Flow cytometry data showing gating strategy and quality control. Representative flow cytometric analysis of detached retina showing the gating strategy to identify microglia ($CD45^{mid}$ $CD11b^{+}$ $P2ry12^{+}$) and macrophage ($CD45^{high}$ $CD11b^{+}$ $P2ry12^{-}$) population. Microglial and macrophage populations were further gated to determine C3aR expression.

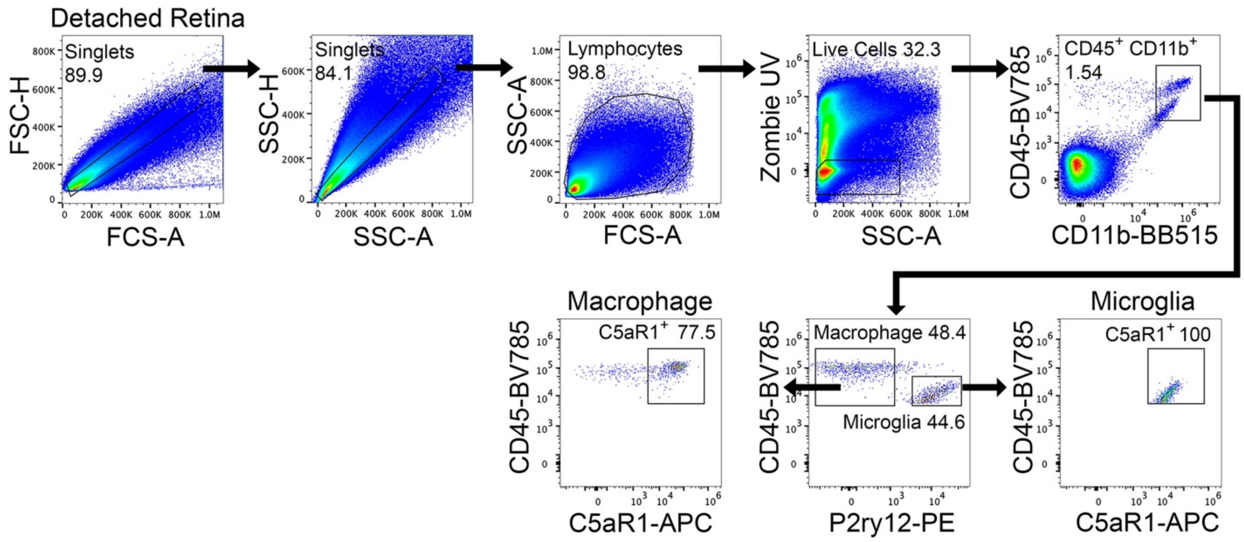

Supplementary figure S2: Flow cytometry data showing gating strategy and quality control. Representative flow cytometric analysis of detached retina showing the gating strategy to identify microglia ($CD45^{mid}$ $CD11b^{+}$ $P2ry12^{+}$) and macrophage ($CD45^{high}$ $CD11b^{+}$ $P2ry12^{-}$) population. Microglial and macrophage populations were further gated to determine C5aR1 expression.
